# Predicting neonatal mortality prior to discharge from hospital in prenatally diagnosed left congenital diaphragmatic hernia

**DOI:** 10.1002/uog.29121

**Published:** 2024-10-24

**Authors:** S. Shinar, A. Otvodenko, D. Kajal, P. P. L. Chiu, S. Lee, P. S. Shah, T. Van Mieghem, Y. Kunpalin, A.‐M. Guerguerian, G. Ryan, N. Abbasi

**Affiliations:** ^1^ Ontario Fetal Centre, Division of Maternal–Fetal Medicine, Department of Obstetrics & Gynaecology Mount Sinai Hospital, University of Toronto Toronto ON Canada; ^2^ Department of Medical Imaging, Women's College Hospital & Mount Sinai Hospital University of Toronto Toronto ON Canada; ^3^ Division of General and Thoracic Surgery, Department of Surgery The Hospital for Sick Children, University of Toronto Toronto ON Canada; ^4^ Maternal–Infant Care Research Centre Mount Sinai Hospital Toronto Ontario Canada; ^5^ Department of Paediatrics, Division of Neonatology, Mount Sinai Hospital University of Toronto Toronto ON Canada; ^6^ Department of Critical Care Medicine The Hospital for Sick Children, University of Toronto Toronto ON Canada

**Keywords:** CDH, congenital diaphragmatic hernia, magnetic resonance imaging, MRI, neonatal mortality, ultrasound

## Abstract

**Objectives:**

To evaluate the association of standardized prenatal imaging parameters and immediate neonatal variables with mortality prior to discharge in infants with isolated left congenital diaphragmatic hernia (LCDH), and to compare the performance of ultrasound‐ and magnetic resonance imaging (MRI)‐based severity grading for the prediction of neonatal mortality.

**Methods:**

This was a retrospective study of infants with prenatally diagnosed isolated LCDH referred to a single tertiary center between 2008 and 2020. Fetuses with right or bilateral congenital diaphragmatic hernia, additional major structural anomaly or known genetic condition, as well as cases that underwent fetal intervention or declined postnatal intervention, were excluded. Ultrasound and MRI images were reviewed retrospectively. Univariable and multivariable analyses were performed, incorporating prenatal and immediate neonatal factors to analyze the association with neonatal mortality prior to discharge, and a prediction calculator was generated. The performance of ultrasound and that of MRI for the prediction of neonatal mortality were compared.

**Results:**

Of 253 pregnancies with fetal CDH, 104 met the inclusion criteria, of whom 77 (74%) neonates survived to discharge. Seventy‐five fetuses underwent both prenatal ultrasound and MRI. On multivariable analysis, observed/expected (o/e) lung‐to‐head ratio and o/e total fetal lung volume were associated independently with neonatal death (adjusted odds ratio, 0.89 (95% CI, 0.83–0.95) and 0.90 (95% CI, 0.84–0.97), respectively), whereas liver position was not. There was no significant difference in predictive performance between using ultrasound and MRI together (area under the receiver‐operating‐characteristics curve (AUC), 0.85 (95% CI, 0.76–0.93)) compared with using ultrasound alone (AUC, 0.81 (95% CI, 0.72–0.90); *P* = 0.19). The addition of neonatal parameters (gestational age at birth and small‐for‐gestational age) did not improve model performance (AUC, 0.87 (95% CI, 0.80–0.95)) compared with the combined ultrasound and MRI model (*P* = 0.22). There was poor agreement between severity assessment on ultrasound and MRI (Cohen's *κ*, 0.19). Most discrepancies were seen among cases deemed to be non‐severe on ultrasound and severe on MRI, and outcomes were more consistent with MRI‐based prognostication.

**Conclusions:**

In fetuses with prenatally diagnosed isolated LCDH, mortality prediction using standardized ultrasound and MRI measurements performed reasonably well. In cases classified as non‐severe on ultrasound, MRI is recommended, as it may provide more accurate prognostication and assist in the determination of candidacy for fetal intervention. © 2024 The Author(s). *Ultrasound in Obstetrics & Gynecology* published by John Wiley & Sons Ltd on behalf of International Society of Ultrasound in Obstetrics and Gynecology.

**CONTRIBUTION**

*What are the novel findings of this work?*

In isolated left congenital diaphragmatic hernia (LCDH), the predictive values of ultrasound and magnetic resonance imaging (MRI) for neonatal mortality prior to hospital discharge are similar. The addition of immediate neonatal parameters did not improve prognostication. Among fetuses with observed/expected (o/e) lung‐to‐head ratio (LHR) > 25% on ultrasound, MRI predicted more severe pulmonary hypoplasia (o/e total fetal lung volume ≤ 35%) in 31% of cases, and the postnatal course was more consistent with MRI prognostication.

*What are the clinical implications of this work?*

Accurate prenatal prognostication for isolated LCDH can be provided to families from midgestation onwards using ultrasound or MRI. In cases deemed to be non‐severe on ultrasound (o/e‐LHR > 25%), MRI should be considered for the more accurate prediction of mortality and may be of additional value in determining candidacy for fetal therapy.

## INTRODUCTION

Congenital diaphragmatic hernia (CDH) is a defect of the diaphragm resulting in herniation of abdominal viscera into the thorax, with an incidence of 1 in 4000 live births^1^. The substantial neonatal morbidity and mortality associated with CDH arise primarily from pulmonary hypoplasia and pulmonary hypertension^2^, ^3^, ^4^, ^5^. The degree of pulmonary hypoplasia may be assessed by estimating lung size on ultrasound and magnetic resonance imaging (MRI) and can be used to predict neonatal mortality^6^, ^7^. Prenatal prognostication is important because it can assist in triaging patients to high‐risk fetal and neonatal centers and identifying candidates for prenatal intervention with fetal endoscopic tracheal occlusion (FETO) to improve survival.

The most extensively studied prenatal imaging‐based prognostic indicator for the prediction of pulmonary hypoplasia in CDH is the ultrasound‐determined observed/expected (o/e) lung‐to‐head ratio (LHR)^8^. o/e‐LHR was found to be an independent sonographic predictor of CDH‐related neonatal mortality^8^, ^9^, ^10^, ^11^ and morbidity^11^, ^12^. Additionally, MRI can be used to assess the total fetal lung volume (TFLV) in three dimensions to obtain the o/e‐TFLV, which is also a significant predictor of mortality in CDH and an important additional tool in CDH prognostication^13^, ^14^, ^15^, ^16^, ^17^. Recent systematic reviews and meta‐analyses have demonstrated that o/e‐LHR on ultrasound, o/e‐TFLV on MRI and the presence and extent of liver herniation are good predictors of neonatal mortality in CDH^10^, ^16^, ^18^, ^19^. Nonetheless, evaluation and comparison of ultrasound and MRI performance for prognostication in CDH are limited by variations in imaging techniques and protocols across sites. As such, the need for standardization in the prenatal assessment of CDH to improve reproducibility and prognostic value has been recognized by the North American Fetal Therapy Network^20^, ^21^ and the European Reference Network for Rare Inherited and Congenital Anomalies^22^. Furthermore, prenatal parameters are limited in predicting neonatal outcome because perinatal factors such as gestational age (GA) at delivery and birth weight may also impact survival^23^, ^24^. Thus, several prediction models have been introduced to facilitate accurate prenatal counseling and prepare families and pediatric teams. Some models are based on prenatal factors^25^, ^26^, ^27^, but the majority are derived from neonatal factors alone^25^, ^27^, ^28^, ^29^, ^30^, ^31^, thereby excluding important prenatal variables, often owing to a lack of standardized measurements or missing prenatal data. An additional limitation of many existing models is inclusion of right CDH (RCDH) in addition to left CDH (LCDH), as well as isolated and non‐isolated CDH, which contributes to an increased mortality risk^8^, ^32^, ^33^, ^34^.

Recognizing the current limitations of prenatal prognostication and risk stratification for prenatally diagnosed CDH, our primary objectives in this study were to evaluate the utility of standardized prenatal imaging parameters and perinatal factors for the prediction of neonatal mortality prior to discharge in fetuses with isolated LCDH and to develop a CDH mortality prediction calculator based on prenatal and perinatal factors. Our secondary objective was to compare the agreement and accuracy of standardized prenatal ultrasound and MRI assessment in the prediction of neonatal mortality prior to discharge in this population.

## METHODS

This was a retrospective study of all fetuses referred with prenatally diagnosed CDH to the Ontario Fetal Centre, Toronto, Canada, between January 2008 and December 2020. The Ontario Fetal Centre is a collaboration between Mount Sinai Hospital and the Hospital for Sick Children in Toronto and serves as a tertiary referral center. It is the only center offering fetal intervention for CDH in Canada. Ethical approval for this study was granted by both hospital institutional review boards (REB #13‐0077‐C and 1000006184).

Fetuses with prenatally detected RCDH or bilateral CDH, additional major structural anomaly or known genetic condition detected pre‐ or postnatally, those that underwent fetal intervention and those for which the parents opted for perinatal palliative care were excluded. Anomalies not considered to affect survival in the presence of normal genetic testing were included (e.g. small ventricular septal defect, mild‐to‐moderate pelviectasis, duplex renal collecting system, unilateral talipes, small branchial cleft cyst, partial agenesis of the corpus callosum, mesenteric cyst or isolated mild ventriculomegaly of ≤ 12 mm). In our institution, all patients with CDH were offered routine genetic testing in the form of karyotyping and, from 2014 onward, chromosomal microarray analysis. Postnatal testing was performed routinely if declined prenatally after 2014; however, prior to this, postnatal testing was at the discretion of the clinician and typically limited to infants with non‐isolated CDH.

Digitally stored ultrasound images were reviewed independently by two experienced fetal medicine specialists (N.A., S.S.) who were blinded to the neonatal outcome. Prenatal prognostication was based on determination of the o/e‐LHR utilizing the ‘trace method’ owing to its higher inter‐rater agreement compared with other methods^35^, ^36^ and the calculator provided by the Tracheal Occlusion To Accelerate Lung Growth trial (www.totaltrial.eu). As the liver may be difficult to distinguish from the bowel and lung sonographically, the stomach position (intra‐abdominal and intrathoracic – anterior, mid or posterior/retrocardiac) was used as a surrogate for intrathoracic liver herniation, with posterior/retrocardiac positioning suggestive of the most significant liver herniation^37^, ^38^, ^39^. Stomach position has also been shown to correlate with liver herniation on MRI^38^ as well as neonatal mortality^37^, ^38^. The severity of pulmonary hypoplasia/CDH was determined by o/e‐LHR and the presence of liver herniation, consistent with previously described algorithms for the prediction of survival in LCDH^8^, ^40^. Specifically, an o/e‐LHR of ≤ 25.0% was defined as severe pulmonary hypoplasia; an o/e‐LHR of 25.1–34.9% irrespective of liver position or 35.0–44.9% with liver herniation was considered moderate hypoplasia; and an o/e‐LHR of ≥ 45.0% irrespective of liver position or 35.0–44.9% without liver herniation was defined as mild hypoplasia. In cases where serial ultrasound exams were available, we analyzed the highest‐quality images that were obtained closest to the time of MRI. This was deemed most appropriate because it was at this time that candidacy for FETO was established. Fetal MRI images were reviewed retrospectively by an experienced fetal medicine radiologist (D.K.) who was blinded to the neonatal outcome. Assessment of o/e‐TFLV, presence of liver herniation, extent of liver herniation (i.e. percentage liver herniation (%LH))^15^, liver‐to‐thoracic volume ratio (LiTR)^14^ and lung‐to‐liver signal intensity ratio (LLSIR)^41^ to evaluate lung maturation were performed retrospectively.

Obstetric and neonatal outcomes collected included GA at delivery, preterm birth before 37 weeks, preterm prelabor rupture of membranes (PPROM) before 37 weeks, GA at PPROM, fetal sex, birth weight, small‐for‐gestational age (SGA) (defined as birth weight < 10^th^ percentile)^42^, first neonatal arterial pH from cord blood, type of ventilation, pneumothorax and details of diaphragmatic repair. During the study period, clinical practice did not change significantly, and the timing of repair generally aligned with the infant's capacity to undergo surgery.

The primary outcome was neonatal death prior to discharge following initial hospitalization after delivery. The association of the primary outcome with prenatal demographic variables, imaging characteristics on ultrasound and MRI, and immediate neonatal factors was evaluated. Mortality prediction models incorporating prenatal imaging characteristics, with and without immediate neonatal variables, were compared for their performance. Ultrasound and MRI predictors of pulmonary hypoplasia were compared for their ability to predict postnatal mortality.

Baseline characteristics and outcomes were compared between survivors and non‐survivors using the chi‐square test or Fisher's exact test for categorical variables and Student's *t‐*test or the Wilcoxon rank‐sum test for continuous variables. Multiple logistic regression models were used to investigate the effect of prenatal imaging characteristics and immediate neonatal factors on survival, and they were adjusted for risk factors and potential confounders identified on univariable analysis. Different CDH prediction models were developed and the area under the receiver‐operating‐characteristics curve (AUC) was estimated to determine the accuracy of these models for mortality prediction. Multicollinearity among the risk factors and potential confounders was assessed using the variance inflation factor (VIF). Additionally, Cohen's κ statistic was utilized to examine the agreement between ultrasound and MRI for severe *vs* non‐severe pulmonary hypoplasia, using cut‐off values of 25% and 35% for ultrasound and MRI, respectively, based on data published previously from our institution^9^. κ‐values were categorized as follows: 0.0–0.20, poor agreement; 0.21–0.40, fair agreement; 0.41–0.60, moderate agreement; 0.61–0.80, good agreement; 0.81–1.0, very good agreement. Statistical analysis was conducted using SAS software version 9.4 (Cary, NC, USA). Two‐sided *P* < 0.05 was considered to indicate statistical significance.

## RESULTS

**Figure 1:**
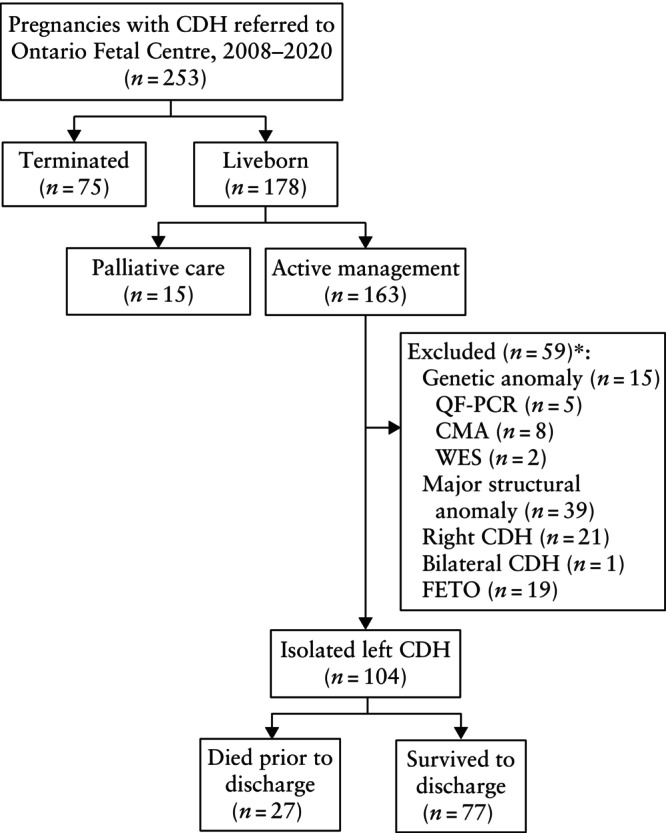
Flowchart summarizing inclusion in study of expectantly managed pregnancies with isolated left congenital diaphragmatic hernia (CDH). *Some cases had more than one reason for exclusion. CMA, chromosomal microarray; FETO, fetal endoscopic tracheal occlusion; QF‐PCR, quantitative fluorescence polymerase chain reaction; WES, whole‐exome sequencing.

During the 13‐year study period, 253 pregnancies were seen at the Ontario Fetal Centre with prenatally confirmed CDH. Of those pregnancies, 30% (*n* = 75) were terminated. Among 178 liveborn infants, 15 (8%) were planned for postnatal palliative care and 163 (92%) received active perinatal care. The majority of infants receiving active postnatal care had LCDH (*n* = 141 (87%)), while 21 (13%) had RCDH and one (0.6%) neonate had bilateral CDH (Figure 1). Additional structural anomalies and/or underlying genetic conditions were detected in 39/163 (24%) pregnancies. FETO was performed in 12% (19/163) of cases. Among fetuses with LCDH, major structural anomalies were demonstrated in 13.5% (19/141) and abnormal genetic results in 15% (11/72 pregnancies that received genetic testing). There were 104 neonates with isolated LCDH who did not undergo FETO and were planned for active management, and these comprised our study cohort. Within this cohort, 27/104 (26%) infants died prior to discharge from hospital. When stratified by o/e‐LHR severity, neonatal mortality was 2% (1/48), 40% (19/48) and 88% (7/8) for mild, moderate and severe pulmonary hypoplasia, respectively.

### Prenatal prediction of mortality prior to discharge from hospital

On univariable analysis (Table 1), earlier GA at diagnosis was associated with mortality (*P* = 0.004); however, there were no significant differences in preterm birth, PPROM or fetal sex between survivors and non‐survivors. The median GA at o/e‐LHR assessment on ultrasound was 24.1 (interquartile range (IQR), 22.0–32.9) weeks, and lower o/e‐LHR (*P* < 0.0001), o/e‐LHR in the severe range (*P* < 0.0001) and intrathoracic posterior/retrocardiac stomach position (*P* = 0.0003) were associated with neonatal death. Seventy‐five (72%) fetuses underwent MRI at a median GA of 27.0 (IQR, 26.0–29.4) weeks. Based on MRI parameters, neonatal mortality was associated with lower o/e‐TFLV (*P* = 0.0001) and increased %LH (*P* = 0.01) and LiTR (*P* = 0.03), but it was not associated significantly with LLSIR (*P* = 0.17). On multivariable analysis incorporating select prenatal imaging parameters with VIF < 3, only o/e‐LHR and o/e‐TFLV were associated independently with neonatal death (adjusted odds ratio (aOR), 0.89 (95% CI, 0.83–0.95) and 0.90 (95% CI, 0.84–0.97), respectively) (Table 2).

**Table 1:** Univariable analysis of prenatal risk factors for neonatal death prior to discharge in isolated left congenital diaphragmatic hernia

Variable	All (*n* = 104)	Survivors (*n* = 77)	Non‐survivors (*n* = 27)	*P* *
GA at diagnosis (weeks)	22 (20–32)	23 (21–32)	21 (19–23)	0.004
PPROM	6 (6)	4 (5)	2 (7)	0.65
PTB < 37 weeks	15 (14)	9 (12)	6 (22)	0.21
Female sex	38 (37)	28 (36)	10 (37)	0.95
Ultrasound parameters				
o/e‐LHR	41.58 ± 13.17	45.32 ± 12.49	30.92 ± 8.48	< 0.0001
o/e‐LHR severity				< 0.0001
Mild	48 (46)	47 (61)	1 (4)	
Moderate	48 (46)	29 (38)	19 (70)	
Severe	8 (8)	1 (1)	7 (26)	
Stomach position				0.0003
Intra‐abdominal, intrathoracic anterior or mid	68 (65)	58 (75)	10 (37)	
Intrathoracic posterior/retrocardiac	36 (35)	19 (25)	17 (63)	
Polyhydramnios	25 (24)	16 (21)	9 (33)	0.19
MRI parameters†				
o/e‐TFLV	42.7 ± 13.8	46.4 ± 12.80	33.1 ± 11.9	0.0001
Liver location				0.051
Intra‐abdominal (‘down’)	42/75 (56)	34/54 (63)	8/21 (38)	
Intrathoracic (‘up’)	33/75 (44)	20/54 (37)	13/21 (62)	
%LH	0 (0–18)	0 (0–11)	17 (0–32)	0.01
LiTR	0 (0–15)	0 (0–12)	11 (0–18)	0.03
LLSIR	2.2 (1.9–2.5)	2.3 (2.0–2.5)	2.2 (1.7–2.5)	0.17

Data are presented as median (interquartile range), *n* (%), mean ± SD or *n*/*N* (%).

* Comparison between survivor and non‐survivor groups was by chi‐square test or Fisher's exact test, as appropriate, for categorical variables and Student's *t*‐test or Wilcoxon rank‐sum test, as appropriate, for continuous variables.

† Only 75 fetuses underwent both ultrasound and magnetic resonance imaging (MRI).

%LH, percentage liver herniation; GA, gestational age; LHR, lung‐to‐head ratio; LiTR, liver‐to‐thoracic volume ratio; LLSIR, lung‐to‐liver signal intensity ratio; o/e, observed/expected; PPROM, preterm prelabor rupture of membranes; PTB, preterm birth; TFLV, total fetal lung volume.

**Table 2:** Multivariable logistic regression analysis assessing association of prenatal risk factors on ultrasound and magnetic resonance imaging (MRI) with neonatal death prior to discharge in isolated left congenital diaphragmatic hernia

Variable	Unadjusted OR (95% CI)	Adjusted OR (95% CI)
Ultrasound		**
GA at diagnosis*	0.38 (0.13–1.10)	1.16 (0.26–5.14)
o/e‐LHR†	0.87 (0.82–0.93)	0.89 (0.83–0.95)
Stomach position‡	5.19 (2.03–13.25)	1.83 (0.58–5.83)
Female sex	1.03 (0.41–2.56)	1.65 (0.50–5.44)
Polyhydramnios	1.91 (0.72–5.03)	1.40 (0.42–4.71)
MRI		††
GA at diagnosis*	0.48 (0.12–1.87)	0.63 (0.10–3.95)
o/e‐TFLV†	0.91 (0.86–0.96)	0.90 (0.84–0.97)
Liver location§	2.76 (0.98–7.81)	0.62 (0.15–2.63)
LLSIR¶	0.41 (0.14–1.22)	0.84 (0.22–3.18)
Female sex	1.34 (0.47–3.83)	2.30 (0.63–8.32)
Polyhydramnios	2.00 (0.70–5.73)	1.42 (0.40–5.12)

* Reference is < 28 weeks.

† Per 1% increase.

‡ Intra‐abdominal, intrathoracic anterior or mid *vs* intrathoracic posterior/retrocardiac.

§ Intra‐abdominal *vs* intrathoracic.

¶ Per 1‐unit increase.

** Odds ratio (OR) adjusted for gestational age (GA) at diagnosis, observed/expected (o/e) lung‐to‐head ratio (LHR) on ultrasound, stomach position on ultrasound, fetal sex and polyhydramnios in 104 fetuses that underwent ultrasound.

†† OR adjusted for GA at diagnosis, o/e total fetal lung volume (TFLV), liver location, lung‐to‐liver signal intensity ratio (LLSIR), fetal sex and polyhydramnios in 75 fetuses that underwent both ultrasound and MRI.

**Figure 2:**
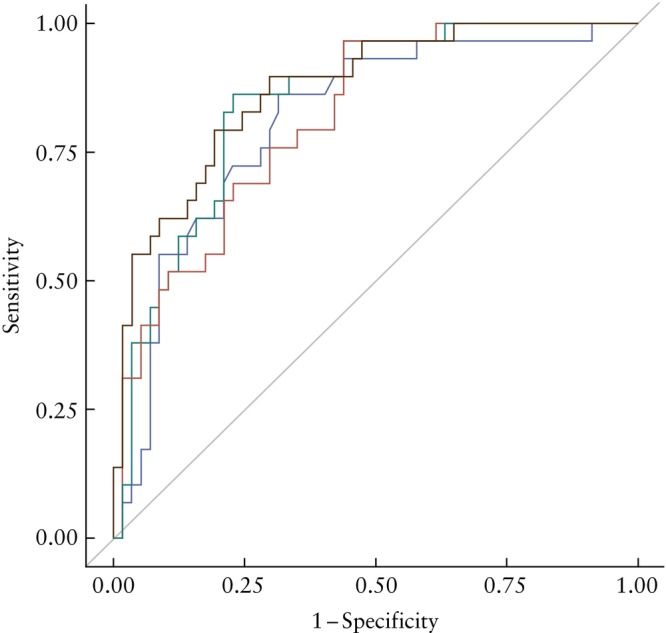
Receiver‐operating‐characteristics curves showing performance of ultrasound parameters, magnetic resonance imaging (MRI) parameters and perinatal factors in predicting neonatal death prior to discharge in isolated left congenital diaphragmatic hernia. 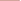, ultrasound‐only model (includes gestational age (GA) at diagnosis, observed/expected (o/e) lung‐to‐head ratio, intrathoracic posterior/retrocardiac stomach position, female sex and polyhydramnios); 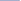, MRI‐only model (includes GA at diagnosis, o/e total fetal lung volume, intrathoracic liver location and female sex); 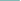, ultrasound + MRI model (includes all variables from ultrasound‐only model and MRI‐only model); 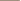, ultrasound + MRI + immediate neonatal factors model (includes all variables from ultrasound + MRI model plus small‐for‐gestational age and GA at delivery < 37 weeks).

The performance of ultrasound alone for predicting neonatal mortality in isolated LCDH when incorporating select prenatal characteristics (GA at diagnosis, o/e‐LHR, intrathoracic posterior/retrocardiac stomach position, female sex and polyhydramnios) was acceptable, with an AUC of 0.81 (95% CI, 0.72–0.90) (Figure 2). The performance of MRI alone when incorporating GA at diagnosis, o/e‐TFLV, liver location (intra‐abdominal *vs* intrathoracic) and fetal sex was similar to that of ultrasound, with an AUC of 0.81 (95% CI, 0.69–0.93) (*P* = 0.94). No significant difference was observed when LLSIR was added to the model (AUC, 0.78 (95% CI, 0.67–0.89); *P* = 0.72). When combining ultrasound and MRI parameters into a single model incorporating both o/e‐LHR and o/e‐TFLV, there was no significant difference in performance (AUC, 0.85 (95% CI, 0.76–0.93)) compared with that of ultrasound alone (*P* = 0.19).

### Neonatal factors associated with mortality

When evaluating immediate neonatal factors, lower birth weight and SGA were associated with death prior to discharge from hospital (*P* = 0.0007 and *P* = 0.004, respectively) (Table 3). No significant differences were seen in GA at delivery, preterm birth before 37 weeks and PPROM between survivors and non‐survivors (Table 1). Use of extracorporeal membrane oxygenation (*P* < 0.0001) and later age at surgical repair (median, 19.5 days *vs* 4 days; *P* = 0.003) were more common among non‐survivors compared with survivors (Table 3). The rate of pneumothorax did not differ significantly between groups.

**Table 3:** Univariable analysis of immediate neonatal risk factors for neonatal death prior to discharge in isolated left congenital diaphragmatic hernia

Variable	Survivors (*n* = 77)	Non‐survivors (*n* = 27)	*P* *
GA at delivery (weeks)	38.9 (38.1–39.9)	39.1 (37.0–39.7)	0.60
Birth weight (kg)	3.2 (2.9–3.5)	2.9 (2.1–3.1)	0.0007
SGA	13 (17)	12 (44)	0.004
Invasive ventilation			< 0.0001
Conventional	42 (55)	2 (7)	
High frequency	31 (40)	18 (67)	
ECMO	4 (5)	7 (26)	
Age at repair (days)	4 (2–9)	19.5 (15–27)	0.003
Patch repair	36 (47)	6/20 (30)	0.18
First cord arterial pH	7.2 ± 0.12	7.0 ± 0.16	< 0.0001
Pneumothorax	8/66 (12)	6/21 (29)	0.09

Data are presented as median (interquartile range), *n* (%), mean ± SD or *n*/*N* (%).

* Comparison between groups was by chi‐square test or Fisher's exact test, as appropriate, for categorical variables and Student's *t*‐test or Wilcoxon rank‐sum test, as appropriate, for continuous variables.

ECMO, extracorporeal membrane oxygenation; GA, gestational age; SGA, small‐for‐gestational age (birth weight < 10^th^ percentile).

On multivariable logistic regression analysis, incorporating prenatal and immediate neonatal parameters (GA at delivery and SGA), the only independent variables associated with mortality were o/e‐LHR on ultrasound (aOR, 0.88 (95% CI, 0.79–0.97)), o/e‐TFLV on MRI (aOR, 0.91 (95% CI, 0.84–0.99)) and SGA (aOR, 9.57 (95% CI, 1.85–49.50)) (Table 4). The mortality prediction model incorporating select prenatal variables (GA at diagnosis, o/e‐LHR, intrathoracic posterior/retrocardiac stomach position on ultrasound, o/e‐TFLV, intrathoracic liver location on MRI, female sex and polyhydramnios) and immediate neonatal variables (GA at delivery and SGA) had an AUC of 0.87 (95% CI, 0.80–0.95), which was similar to the ultrasound‐only model (difference in AUC (ΔAUC) = 0.06; *P* = 0.06) and to the prenatal model combining ultrasound and MRI (ΔAUC = 0.03; *P* = 0.22) (Figure 2). A prediction calculator for clinical use was created on the basis of this model using prenatal variables with or without immediate neonatal variables where available (Appendix S1).

**Table 4:** Multivariable logistic regression analysis assessing association of prenatal and immediate neonatal risk factors with neonatal death prior to discharge in isolated left congenital diaphragmatic hernia

Variable	VIF	Unadjusted OR (95% CI)	Adjusted OR (95% CI)*
GA at delivery†	1.80	0.83 (0.69–1.004)	1.23 (0.86–1.74)
PPROM	1.77	5.05 (1.37–18.56)	11.91 (0.30–471.29)
SGA	1.05	3.26 (1.16–9.17)	9.57 (1.85–49.50)
Female sex	1.03	1.32 (0.52–3.37)	2.70 (0.63–11.55)
o/e‐LHR‡	1.75	0.89 (0.84–0.95)	0.88 (0.79–0.97)
o/e‐TFLV‡	2.21	0.91 (0.87–0.96)	0.91 (0.84–0.99)
Liver location§	1.82	3.88 (1.47–10.25)	0.69 (0.13–3.62)

* Adjusted for gestational age (GA) at delivery, preterm prelabor rupture of membranes (PPROM) < 37 weeks, small‐for‐gestational age (SGA) (birth weight < 10^th^ percentile) based on sex‐specific birth‐weight charts^42^, fetal sex, observed/expected (o/e) lung‐to‐head ratio (LHR) on ultrasound, o/e total fetal lung volume (TFLV) on magnetic resonance imaging (MRI) and liver location on MRI.

† Per 1‐week increase.

‡ Per 1% increase.

§ Intra‐abdominal *vs* intrathoracic.

OR, odds ratio; VIF, variance inflation factor.

### Performance of ultrasound *vs* MRI for prediction of mortality

The agreement between o/e‐LHR and o/e‐TFLV for severe pulmonary hypoplasia was compared. Based on local data, o/e‐LHR ≤ 25% and o/e‐TFLV ≤ 35% were defined as severe LCDH, with survival of approximately 25%^9^, which was confirmed in this study (Figure S1). Discrepancy in severe LCDH prediction was defined as any case for which one of o/e‐LHR or o/e‐TFLV was consistent with severe pulmonary hypoplasia while the other estimated milder hypoplasia (e.g. a LCDH fetus with o/e‐LHR on ultrasound of > 25% and o/e‐TFLV on MRI of ≤ 35%). The median interval between ultrasound and MRI assessment was 2.86 (IQR, 1.29–5.29) weeks. Among cases of isolated LCDH undergoing both MRI and ultrasound, a discrepancy was seen in 29% (22/75) of cases. Agreement for severe or non‐severe CDH as determined by ultrasound (≤ 25% and > 25%, respectively) and MRI (≤ 35% and > 35%, respectively) was poor, with a Cohen's κ of 0.19 (95% CI, 0.02–0.40).

**Figure 3:**
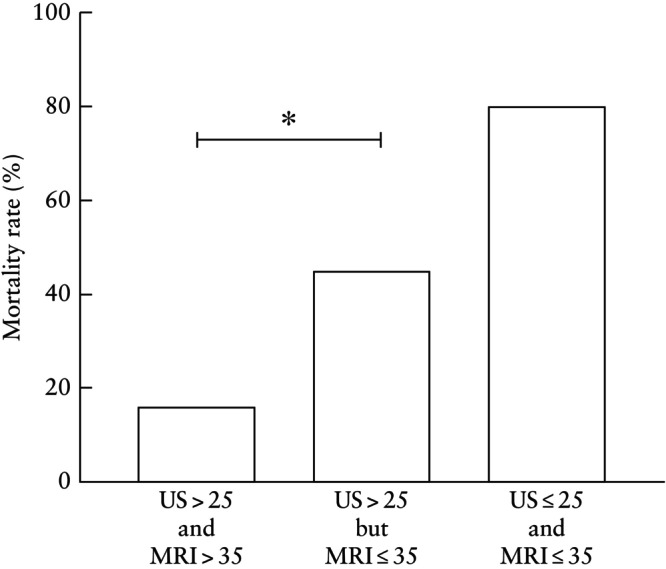
Neonatal mortality rates in 75 cases of isolated left congenital diaphragmatic hernia with concordant or discordant prediction of severe pulmonary hypoplasia on ultrasound (US) (observed/expected lung‐to‐head ratio ≤ 25% (US ≤ 25) or > 25% (US > 25)) and magnetic resonance imaging (MRI) (observed/expected total fetal lung volume ≤ 35% (MRI ≤ 35) or > 35% (MRI > 35)). *P = 0.009.

Among 71 fetuses with o/e‐LHR > 25%, MRI predicted more severe pulmonary hypoplasia (o/e‐TFLV ≤ 35%) in 22 (31%) cases. Mortality was significantly higher among these discordant cases compared with those with concordant o/e‐TFLV in the non‐severe range (> 35%) (45% (10/22) *vs* 16% (8/49); *P* = 0.009) (Figure 3). Of note, all cases of severe CDH with o/e‐LHR ≤ 25% had concordant o/e‐TFLV (i.e. ≤ 35%), and all cases of mild CDH with o/e‐LHR > 45% had concordant o/e‐TFLV (i.e. > 35%).

## DISCUSSION

In isolated LCDH, o/e‐LHR, o/e‐TFLV and SGA were associated independently with neonatal mortality prior to discharge. Ultrasound and MRI using standardized imaging techniques performed similarly in predicting mortality, either alone or in combination. The inclusion of immediate neonatal parameters did not improve predictive performance further. There was poor agreement between ultrasound and MRI for severe pulmonary hypoplasia in LCDH. Discordance between ultrasound and MRI for the prediction of disease severity was seen in 29% of cases, specifically those classified as non‐severe on ultrasound (o/e‐LHR > 25%). Among infants for whom MRI suggested severe hypoplasia (o/e‐TFLV ≤ 35%) but ultrasound suggested non‐severe hypoplasia (o/e‐LHR > 25%), postnatal outcomes were more consistent with MRI prognostication, suggesting that MRI is superior to ultrasound evaluation in these cases.

### Predictors of mortality

Consistent with previous studies^8^, ^18^, ^43^, ^44^, an association between CDH mortality and both o/e‐LHR and o/e‐TFLV was observed in our cohort; these were the only imaging parameters associated independently with neonatal mortality. Evaluation of lung maturation with LLSIR has also been proposed for CDH prognostication^41^, ^45^. However, in our cohort, LLSIR was not associated independently with mortality. Additionally, in contrast to previous series^14^, ^15^, ^18^, ^19^, ^46^, ^47^, ^48^, liver location, %LH and LiTR were not associated independently with CDH mortality, probably because these parameters correlate strongly with o/e‐LHR and o/e‐TFLV. Although prior studies have demonstrated an association between prematurity and CDH mortality^24^, ^49^, ^50^, in our series, PPROM rates and GA at delivery did not differ between survivors and non‐survivors, presumably owing to the low rate of prematurity overall in the absence of fetal intervention. In the perinatal period, SGA was associated independently with mortality, as demonstrated previously^28^, ^51^. Lastly, in contrast to a recent study that demonstrated that female sex is a risk factor for mortality in CDH, we found no such association^52^.

### CDH mortality prediction models in the literature

A number of prediction models for CDH mortality have been published, with the majority incorporating postnatal parameters alone^28^, ^31^, ^51^, ^53^, ^54^, ^55^, ^56^, ^57^, others using prenatal parameters only^10^, ^18^, ^25^, ^26^ and some combining both^27^, ^29^, ^53^. When several postnatal prediction models were compared across the Canadian Pediatric Surgical Network^51^, ^54^, ^55^, that of the CDH Study Group performed best, with an AUC of 0.85^58^, which is similar in performance to our prenatal models. Similar to our work, a Japanese study generated a mortality prediction model for isolated LCDH incorporating prenatal and neonatal parameters, with an AUC of 0.94^27^. However, this model utilized non‐standardized imaging measurements and included FETO patients. The strength of our model lies in its exclusion of factors known to affect survival (additional anomalies, RCDH and fetal intervention), its use of standardized prenatal imaging parameters, and its integration of postnatal factors readily available within the first minutes after birth.

### Ultrasound *vs* MRI for mortality prediction in CDH

MRI is increasingly used in conjunction with ultrasound for CDH prognostication. Several studies have demonstrated an association of CDH mortality with o/e‐TFLV ≤ 25–35% and liver herniation on MRI^14^, ^15^, ^17^, ^18^, ^47^, ^48^. However, studies evaluating discordance between ultrasound and MRI for CDH prognostication are sparse. In one series of 42 isolated LCDH cases, 40% had discrepant severity classification based on o/e‐LHR determined by ultrasound and o/e‐TFLV determined by MRI, with nearly 75% of these cases demonstrating more severe estimates of pulmonary hypoplasia on MRI^59^. The most pronounced discrepancy in our study appeared in cases in which o/e‐LHR on ultrasound was > 25%, among which MRI measured smaller lung volumes in approximately one‐third of cases. Notably, postnatal outcomes were more consistent with MRI prognostication. This discrepancy cannot be explained by the measurement methods, as the trace method was utilized for both modalities, or by the timing of MRI, since the median interval between ultrasound and MRI was 3 weeks. Instead, inconsistencies may be related to the varying contribution of the ipsilateral lung to TFLV^60^. These findings also suggest that MRI may be particularly important for prognostication when ultrasound classifies CDH as non‐severe (i.e. o/e‐LHR > 25%). In contrast, among cases deemed to be severe on ultrasound (o/e‐LHR ≤ 25%), MRI was always concordant. These data are consistent with those of previous studies comparing MRI and ultrasound, which show that MRI has a greater predictive value for CDH mortality compared with ultrasound^16^, ^48^. Our findings also have implications for the refinement of FETO selection criteria. In addition to offering FETO for all cases of LCDH with o/e‐LHR ≤ 25%^61^, we advocate that FETO be considered in fetuses with o/e‐LHR > 25% and o/e‐TFLV on MRI ≤ 35%, owing to the higher risk of mortality than that predicted by ultrasound alone in this subgroup.

### Strengths and limitations

One of the major strengths of our study is its assessment of the prognostic performance of standardized techniques of lung‐area measurement. Furthermore, all images were reanalyzed by experienced fetal medicine imaging specialists blinded to the neonatal outcomes. Additionally, we incorporated only simple and immediate neonatal variables to predict postnatal outcome, making our models more generalizable. Lastly, our comparison of ultrasound and MRI included a relatively large number of cases for which standardized imaging data and neonatal outcome were available.

This study is limited by its retrospective nature. Given the sample size, we could not internally validate our model. Genetic testing was not done routinely and very few cases underwent next‐generation sequencing. Finally, our prediction models are applicable only to patients managed with standard postnatal care and not undergoing fetal intervention. However, irrespective of prenatal therapy, our models are relevant for initial counseling and delivery planning once isolated LCDH has been diagnosed.

### Conclusions

Among fetuses with prenatally diagnosed isolated LCDH, prenatal models with standardized ultrasound and MRI measurements performed reasonably well, with no significant difference afforded by incorporating immediate neonatal parameters. As such, prognostication can be offered to families of affected pregnancies from midgestation onwards, which may help in patient selection for fetal procedures. Nearly one‐third of cases deemed to be non‐severe on ultrasound may be reclassified with severe pulmonary hypoplasia on MRI, with greater mortality than expected by ultrasound alone. In this population, MRI is recommended for more precise prognostication and may be of additional value in determining candidacy for fetal therapy.

## Supporting information


**Appendix S1** Calculator for prediction of neonatal mortality prior to discharge in isolated left congenital diaphragmatic hernia

**Figure S1** Correlation plot for observed/expected (o/e) lung‐to‐head ratio (LHR) and o/e total fetal lung volume (TFLV).

## Data Availability

The data that support the findings of this study are available on request from the corresponding author. The data are not publicly available due to privacy or ethical restrictions.
