# Supplementary material for: Predicting neonatal mortality prior to discharge from hospital in prenatally diagnosed left congenital diaphragmatic hernia
Source: Ultrasound Obstet Gynecol. 2024 Oct 24;64(6):746–54. doi: 10.1002/uog.29121 (PMC11609950; doi:10.1002/uog.29121)

**Figure S1** Correlation plot for observed-to-expected (o/e) lung-to-head ratio (LHR) and o/e total fetal lung volume (TFLV)


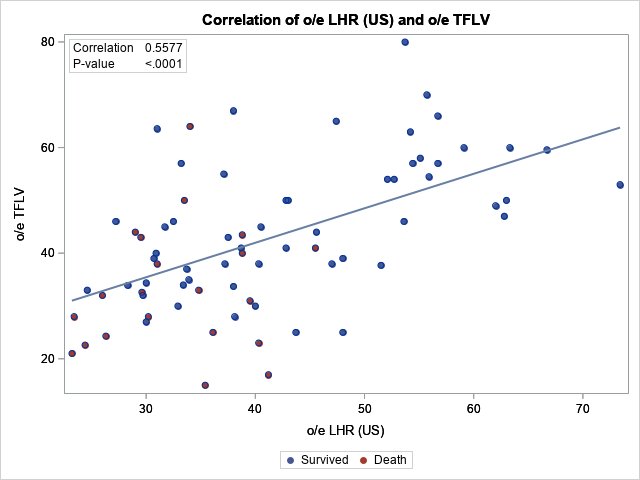


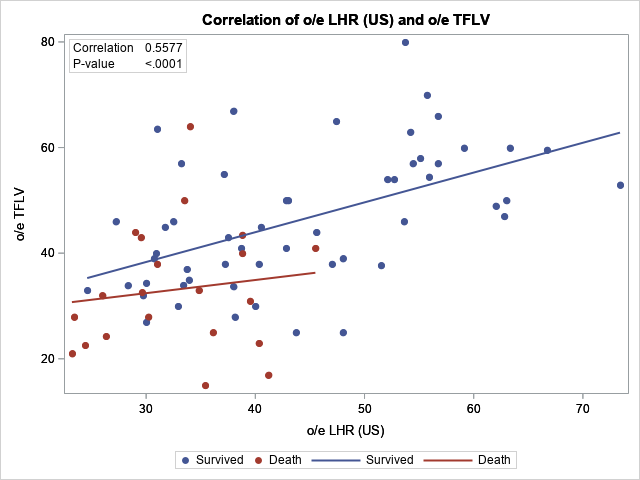

Supplement: Supplementary file 2 — Figure S1 Correlation plot for observed/expected (o/e) lung‐to‐head ratio (LHR) and o/e total fetal lung volume (TFLV). [file UOG-64-746-s001.docx]
